# Origins of Aortic Coarctation: A Vascular Smooth Muscle Compartment Boundary Model

**DOI:** 10.3390/jdb13020013

**Published:** 2025-04-18

**Authors:** Christina L. Greene, Geoffrey Traeger, Akshay Venkatesh, David Han, Mark W. Majesky

**Affiliations:** 1 Heart Center, Seattle Children’s Hospital, Seattle, WA 98112, USA; christina.greene@seattlechildrens.org; 2 Department of Surgery, School of Medicine, University of Washington, Seattle, WA 98105, USA; 3 Norcliffe Foundation Center for Integrated Brain Research, Seattle Children’s Research Institute, Seattle, WA 98101, USA; geoffrey.traeger@seattlechildrens.org; 4 Center for Developmental Biology & Regenerative Medicine, Seattle Children’s Research Institute, Seattle, WA 98101, USA; han@uchc.edu; 5 Department of Medicine, School of Medicine, University of Washington, Seattle, WA 98105, USA; akshay.venkatesh@seattlechildrens.org; 6 Department of Cell Biology & Center for Vascular Biology, University of Connecticut Health Center, Farmington, CT 06030, USA; 7 Department of Pediatrics, School of Medicine, University of Washington, Seattle, WA 98105, USA

**Keywords:** ductus arteriosus, congenital heart disease, lineage, neural crest

## Abstract

Compartment boundaries divide the embryo into segments with distinct fates and functions. In the vascular system, compartment boundaries organize endothelial cells into arteries, capillaries, and veins that are the fundamental units of a circulatory network. For vascular smooth muscle cells (SMCs), such boundaries produce mosaic patterns of investment based on embryonic origins with important implications for the non-uniform distribution of vascular disease later in life. The morphogenesis of blood vessels requires vascular cell movements within compartments as highly-sensitive responses to changes in fluid flow shear stress and wall strain. These movements underline the remodeling of primitive plexuses, expansion of lumen diameters, regression of unused vessels, and building of multilayered artery walls. Although the loss of endothelial compartment boundaries can produce arterial–venous malformations, little is known about the consequences of mislocalization or the failure to form SMC-origin-specific boundaries during vascular development. We propose that the failure to establish a normal compartment boundary between cardiac neural-crest-derived SMCs of the 6th pharyngeal arch artery (future ductus arteriosus) and paraxial-mesoderm-derived SMCs of the dorsal aorta in mid-gestation embryos leads to aortic coarctation observed at birth. This model raises new questions about the effects of fluid flow dynamics on SMC investment and the formation of SMC compartment borders during pharyngeal arch artery remodeling and vascular development.

## 1. Introduction

**Figure 1:**
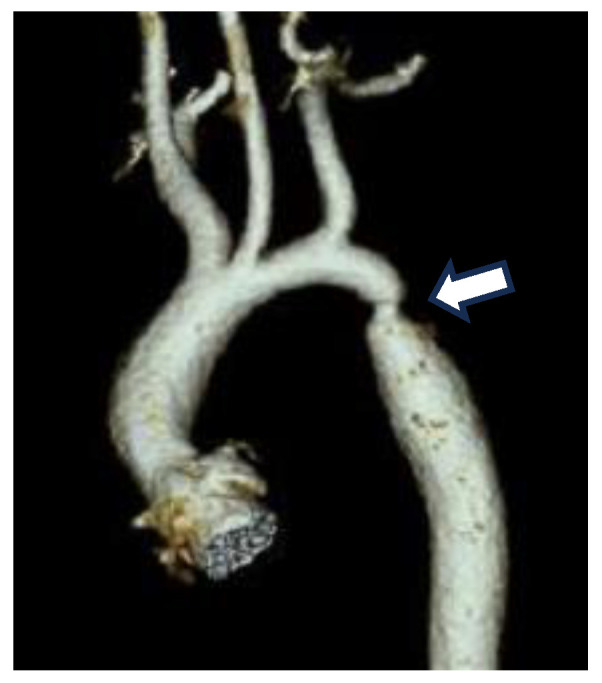
CT image of a constriction site (arrow) in the region where the DA joins the descending AO in a neonatal patient with CoA.

Coarctation of the aorta (CoA) is a form of congenital heart disease that is characterized by a structural narrowing of the aorta that restricts blood flow and thereby impairs normal growth and development of newborns (Figure 1). Blood flow impairment is the result of a constriction of the descending thoracic aorta (AO) usually near the ductal ostia and is thought to be due to vascular smooth muscle cells (SMCs) from the ductus arteriosus (DA) being mislocalized within the wall of the AO. CoA is one of the most frequent presentations of congenital heart disease (CHD) and the most common congenital defect of the aorta, affecting 1 per 2500 live births [1,2]. First diagnosed over two hundred years ago, CoA has been surgically repaired for the last 50 years, yet we have little understanding of the underlying pathobiology. Genome sequencing of CoA and other CHD patient cohorts over the last decade has identified a handful of associated genes, but this effort has failed to establish a unifying mechanism for CoA [3,4,5,6,7,8].

## 2. Development of the Ductus Arteriosus

**Figure 2:**
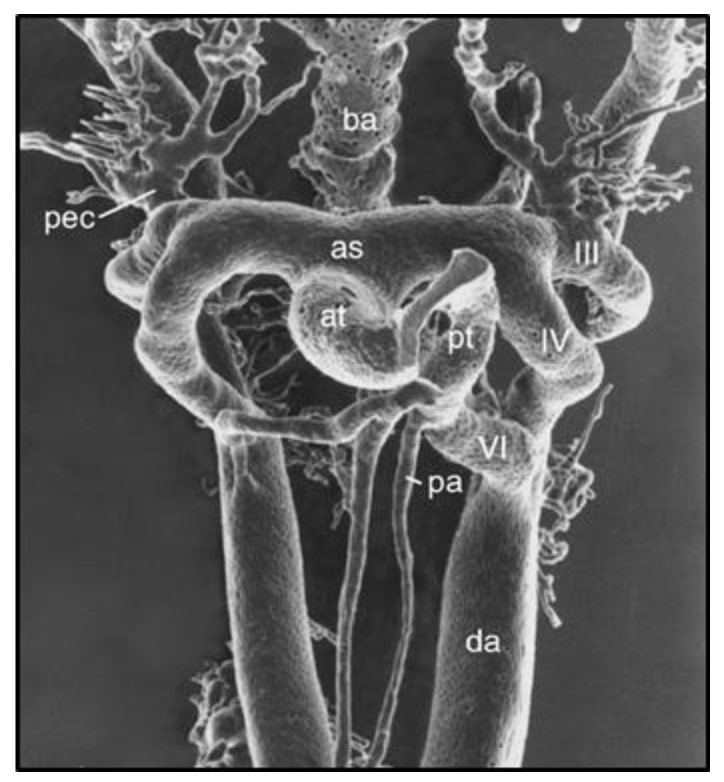
Vascular cast of pharyngeal arch arteries (PAAs) in an E12.0 mouse embryo visualized by scanning electron microscopy. Note the close proximity of PAA IV and VI during the period of SMC investment and the flow-dependent remodeling of the PAA complex. Reprinted with permission from [9].

**Figure 3:**
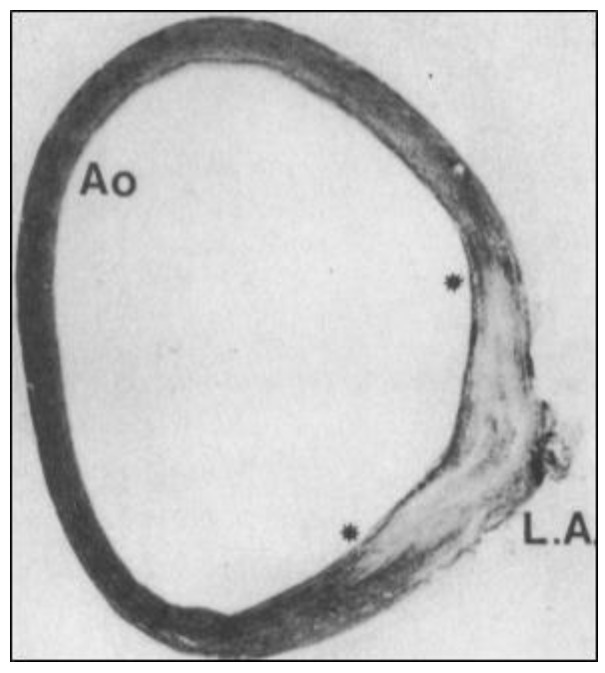
“Fish-tail-like” insertion junction (between the asterisks) of human ductus arteriosus SMCs (LA) into the AO media (reprinted with permission from [19]). Note the absence of organized elastic fibers in the ductal insertion. LA—ligamentum arteriosum.

In searching for the origins of CoA, one must look to the formation and remodeling of the pharyngeal arch arteries (PAAs) in mid-gestation embryos. This is because the DA arises from the flow-sensitive remodeling of the 6th PAA in vascular development [9,10] (Figure 2) and because CoA can be detected by echocardiography in utero [11,12]. Although anatomically continuous with the aortic media, DA-SMCs in the ductal media do not normally extend beyond their junction with the descending thoracic aorta [9,13,14] (Figure 3). As the 6th PAA begins to assemble its complement of SMCs, two types of SMC progenitor cells are present in the pharyngeal arch complex: cardiac neural crest (CNC) and second heart field (SHF) [15]. Endothelial cells of both the 4th arch and 6th arch arteries are derived from SHF progenitors [16]. However, fate-mapping studies have shown that medial SMCs of the 6th PAA originate exclusively from cardiac neural crest cells (CNCs) [17]. In contrast, medial SMCs in the dorsal aorta at the level of the 6th PAA junction arise from paraxial mesoderm (PM) [18]. Therefore, a border is formed at the interface between CNC-derived SMCs in the 6th PAA and PM-derived SMCs in the dorsal aorta. Elzenga et al. described the normal DA-AO junction as a “fish-tail-like” insertion of DA-SMCs within the AO media involving up to one-third of the AO circumference in a newborn infant [19] (Figure 3). The formation of an insertion junction, therefore, means that the DA-SMC interface with AO-SMCs does not exactly correspond to the anatomical landmark of a DA (or later, ligamentum arteriosum—LA) connection to the AO. Nevertheless, the juxtaposition of CNC-derived DA-SMCs and PM-derived AO-SMCs appears to result in a SMC-origin-dependent border that, in effect, acts as a sorting mechanism keeping DA-SMCs confined to the limits of the ductal insertion and maintaining AO-SMCs in a separate anatomical compartment [19]. The necessity of a sorting mechanism becomes evident when the baby takes its first breath at birth. The loss of placental-derived prostaglandins together with an increase in arterial oxygen tension resulting from nascent blood flow to the lungs initiates closure of the DA, a critical event required for adequate perfusion of the now functioning lungs [14,20,21]. The directly adjacent AO-SMCs in the aortic media exhibit none of these oxygen-dependent closure activities. While the DA closes, the AO media continue to increase circumferential growth in proportion to the increasing cardiac output required for postnatal growth of the neonate. The mechanisms that specify 6th arch SMCs to acquire a unique ductal SMC identity while, in close proximity (Figure 2, note the proximity of IV to VI PAAs to each other), dorsal aorta SMCs acquire an entirely different identity and developmental trajectory remain an intriguing mystery yet to be solved.

## 3. Compartment Boundaries in Embryonic Development

The formation of tissue boundaries that divide the embryo into compartments with distinct cell fates is one of the most fundamental processes in development [22,23,24,25,26,27]. For example, compartment boundaries separate adjacent rhombomeres during hindbrain development [28,29,30], somites during axial patterning [31,32,33], septation of the cardiac interventricular septum [34,35], pattern dorsal–ventral domains of the limb bud [36,37], and delineate imaginal discs during development and regeneration in *Drosophila* [26,38]. Compartment boundaries are formed when cells with different identities encounter each other [34,35,38,39]. A lack of mixing at such borders is a direct result of compartment-specific transcriptomes and the maintenance of different cell identities and cell-fate potentials. Studies of the *Drosophila* embryo have provided a conceptual framework for morphogenesis that includes segmentation, the acquisition of cell identity, compartmentation, and pattern formation [39,40]. An important principle that has emerged is that the early embryo is organized into lineage blocks called compartments [38]. Two different lineage blocks that form adjacent to each other are separated by a compartment border that is maintained as a function of different cell identities on either side of the border [38]. If an imaginal disc compartment border is disrupted, for example, then the two compartments regenerate the missing cells, reestablish compartment-specific cell identity, and reorganize the border between them [41]. What mechanisms actually produce compartment borders have been the subject of much investigation [39]. These studies have produced three general models: the differential cell adhesion model [42,43,44], the actin-myosin cortex contraction model [39,45], and the contact repulsive model focused on heterotypic cell–cell contacts [25,27].

## 4. Compartment Boundaries in Vascular Development

In the vascular system, three types of developmental borders have been identified. One is the boundary separating arterial and venous endothelial cells [46,47,48] or arterial and venous SMCs [47,49,50] marked by guidance molecules that mediate contact repulsive signaling between adjacent heterotypic cells. A second type of border separates the aortic root endothelium of second heart field origin [16] from the coronary artery endothelium of sinus venosus origin [51]. This represents an artery–artery EC boundary and, once established, was found to be stable out to at least postnatal day 28 in a mouse model [51]. A third type of compartment boundary separates SMCs of different developmental origins in a common artery wall [17,52,53,54,55,56,57]. Although multiple descriptions of these SMC lineage-specific compartments have been made in the studies cited above and elsewhere, little is known about SMC compartment borders themselves, the molecules and mechanisms that produce these borders, the compartmentalization of SMC functions they maintain, or the consequences of a loss of border function in vascular dysmorphogenesis and disease.

Vascular SMCs from different embryonic origins are products of different lineage histories [52]. Each step in a cell’s progressive acquisition of cell type identity in development is recorded in its epigenome as “footprints” of lineage history [52,58,59]. This molecular memory is represented by patterns of DNA methylation, differences in histone modification, or changes in chromatin accessibility that act together to establish and maintain cell-type-specific profiles of gene expression. If SMCs of different origins and lineage histories are segregated into SMC-origin-specific compartments, these compartments may respond differently to identical stimuli presented systemically (serum cholesterol, high blood pressure, or germ-line mutation). While such responses may be appropriate during vascular development, this differential-response property in adult artery walls may provide the substrate for disease-promoting activity that, if sustained, may manifest as SMC-compartment-dependent vascular disease.

For example, Marfan syndrome patients are at increased risk of developing aortic root and ascending aortic aneurysms [57]. At these high-pressure, high-blood-flow aortic positions, medial SMCs are derived from two embryonic origins, i.e., CNC-SMCs and SHF-SMCs. These two types of aortic SMCs segregate into distinct inner (CNC-SMCs) and outer (SHF-SMCs) domains in the aortic media [54]. Using a well-characterized mouse model of Marfan syndrome carrying a germ-line fibrillin-1 variant allele (Fbn1^C1039G/+^), Nakamura et al. showed that integrin-αv was selectively overexpressed in SHF-SMCs, but not adjacent CNC-SMCs, in ascending aorta in vivo [60]. They also reported that the signaling pathways downstream of αv-integrin including focal adhesion kinase, phospho-Akt^Thr308^, and mechanistic target of rapamycin complex-1/mTORC1 were selectively activated in SHF-SMCs. The treatment of Marfan mice with the αv-integrin antagonist GLPG0187 reduced elastin fragmentation and aneurysm growth, consistent with the SHF-SMCs selectively driving aortic aneurysm pathogenesis in these mice [60].

## 5. Repulsive Guidance Molecule Signaling

Drawing clues from compartment boundary studies in *Drosophila* and other model organisms [25,61,62], we will discuss the lessons learned with a particular focus on the DA-SMC junction with AO-SMCs in the proximal descending thoracic aorta. Chemo-repulsive or contact-repulsive signaling are key border-forming mechanisms employed in embryonic development [50,63,64]. Contact-repulsive guidance molecules are expressed by CNC-derived SMC progenitors enabling them to precisely navigate to the 6th PAA during vascular development [50,65,66,67,68,69,70,71]. Likewise, the migration of paraxial-mesoderm-derived AO-SMC progenitors to the dorsal aorta is also guided by repulsive signaling [31]. During neural crest cell migration in Xenopus embryos, the homophilic calcium-dependent cell–cell adhesion molecule cadherin-11 was found to be necessary for the contact inhibition of movement [72]. This may reflect the cooperative activity of cadherin 11-dependent cell–cell adhesion to form close contacts between adjacent cells so that contact-repulsive molecules are efficiently engaged by those cells [73]. That engagement may initiate the contact-mediated collapse of cell protrusions oriented in the direction of migration [74]. A similar role for N-cadherin in CNC migration has been reported [75]. Upon the contact of DA-SMCs with AO-SMCs, we suggest that the engagement of contact-repulsive guidance molecules stops the migration of both types of vascular SMCs, initiates border-dependent cytoskeletal reorganization [76], and creates a barrier that prevents the mixing of DA-SMCs and AO-SMCs. The abruptness of the DA-AO SMC border [77] (Figure 3) implies a boundary-forming mechanism similar to that which separates arterial and venous endothelial cells in early vascular development [46,48,78,79]. The report of a Semaphorin-3D (*SEMA3D)* loss of function mutation in human CoA is consistent with this hypothesis [6].

It is instructive to consider SMC border formation at another site in the developing vascular system. Lineage- and fate-mapping studies in mouse embryos have shown that SMCs in the aortic root are derived from progenitor cells of either cardiac neural crest or second heart field origin [80]. Sawada et al. showed that these two SMC origins reside in distinct spatial domains in the ascending thoracic aorta with little or no detectable mixing [54]. Cardiac neural-crest-derived SMC progenitors migrate into the pharyngeal arch complex and reach the 4th arch artery wall earlier than second-heart-field-derived SMC progenitors and form the first few inner SMC layers of the aortic root and ascending aorta [80]. Slightly delayed in time, second-heart-field-derived cells also migrate to the developing aorta and constitute the major SMC type in the aortic root, forming the outer medial SMC layers of the ascending aorta [17,53,54,80]. These two types of aortic SMCs express similar levels of SMC contractile marker genes and cytoskeletal genes, but they retain SMC-lineage-specific differences in TGF-β-receptor-dependent growth and transcriptional responses [81,82], baseline gene expression patterns [57,83], and accessible chromatin sites identified by ATAC-seq analysis [84]. Likewise, SMCs from different embryonic origins express different levels of extracellular matrix genes in vivo [85] and in vitro [57].

Important clues about compartment boundary formation in blood vessels can be derived from studies of heart development. The establishment of left and right sides of the interventricular septum (IVS) is necessary for proper patterning of the developing heart [86]. The loss of left/right patterning in the IVS during heart development results in ventricular septal defects (VSDs), a commonly observed congenital heart defect (CHD). The transcription factor *Tbx5* plays a critical role in directing the formation of a compartment border between the two sides of the IVS [35]. Single-cell analysis of the gene expression profiles in *Tbx5* mutant hearts showed downregulation of two important guidance molecules, i.e., Slit2, a ligand for the guidance receptor Robo1, and Netrin1, a ligand for the guidance receptor Unc5b. The reduced expression of Slit2 and Netrin1 was accompanied by the loss of the IVS compartment boundary, expansion of cells into the IVS that are normally restricted to trabecular myocardium, and an increased incidence of CHDs [35]. These important findings suggest the fundamental principles of morphogenesis that are likely to appear elsewhere in cardiovascular development, including the compartment boundary separating DA-SMCs from AO-SMCs in developing PAAs.

## 6. Remodeling of the Pharyngeal Arch Artery Complex

Endothelial cells: Developing blood vessels are highly sensitive to blood flow variations [15,87,88,89,90,91]. Studies in multiple species have shown that changes in blood flow rates are major morphogenic forces acting on endothelial cells, directing their growth, migration, and cell–cell interactions during vascular development [87,88,89,92,93,94,95]. Fluid shear stress forces acting on the endothelial cell surface regulate the assembly of endothelial cell junctions, cytoskeletal organization, polarity, and arterial–venous identity [87,90,91,96,97]. Endothelial cells in developing arteries sense changes in blood flow by multiple shear-stress-sensing mechanisms [96,97,98,99,100,101]. One is a cell surface complex of Cadherin-5/VE-cadherin; PECAM-1; and VEGFR2 [96]. Shear-stress-mediated activation of this complex stimulates phosphatidylinositol-3 kinase and AKT/protein kinase-B pathways, leading to activation of endothelial cytoskeletal and matrix-adhesive adaptive responses. A second shear stress force transducer is a complex of plexin D1, neuropilin1, and VEGFR2 [98]. Plexin D1 is a guidance receptor of the semaphorin family that can also act as a mechanosensitive force receptor in endothelial cells. Through a reversible change in conformation, plexin D1 can either function as a contact-repulsive guidance molecule or a shear-stress-responsive force receptor [98]. In addition to these mechanoresponsive complexes, ECs also sense shear stress forces with G-protein-coupled receptors, including sphingosine-1-phosphate receptor-1 [99]; ion channels, including Piezo1 [100]; and cilia acting via polycystin-1 and Polaris to promote Ca2+ influx and nitric oxide release [101].

The complex remodeling of the PAAs is, in large part, determined by developmental changes in cardiac function, blood flow dynamics, and endothelial shear stress forces [10,91,93]. Using fluorescent dye injections, pulsed Doppler velocity recordings, and micro-CT scans, Wang et al. showed that a shift occurs in flow velocity and wall shear stress (WSS) in the PAA complex between stages HH18 and HH24 in chick embryos [102]. That shift is from a higher WSS in PAA-3 at HH18 to a higher WSS in PAA-4 at HH24 [89,102,103,104]. These developmental shifts in flow velocity and WSS are recorded as corresponding diameter changes in the respective PAAs during the remodeling period [89,102,103].

There is considerable evidence that defects in heart or valve development and the resultant changes in blood flow rate, flow distribution, and endothelial shear stress responses during cardiovascular development produce congenital heart defects [104,105,106]. There is also evidence that both endothelial cells and SMCs are surprisingly motile within the vessel wall during the early stages of vascular development [95,107]. It therefore follows that the junction of the 6th PAA ostia with the dorsal aorta or the investment of this vessel with SMCs may be sensitive to disturbances in normal blood flow dynamics in this critical period of PAA remodeling.

Smooth muscle cells: In response to activated shear-stress-mediated mechanotransduction pathways, ECs release bioactive growth factors and cytokines that play critical roles in the recruitment and differentiation of smooth muscle progenitor cells [108,109,110]. One pathway that functions in the earliest stages of SMC investment in zebrafish and mammalian arteries is the autocrine CXCL12 stimulation of its receptor, CXCR4, on ECs, leading to the increased production of PDGFB, a well-known chemoattractant and mitogen for SMC progenitor cells [109]. PDGFB and its receptor, PDGFRβ, have been shown by loss-of-function experiments to be required for the initial stages of tunica media formation in multiple arteries of mouse and zebrafish embryos [111,112]. It is well-known that the organization of a SMC-containing tunica media is associated with a shift in endothelial phenotype to a more mature, less proliferative state. A recent study by Lee et al. provides evidence that a critical role in this coordinate transition is played by CXCL11-CXCR3 signaling [110]. The inhibition of endothelial-specific CXCR3 signaling produces the loss of pericyte association with endothelial tubes in vitro and in vivo, as well as the proliferative expansion of the vasculature and impairment in mature EC junction formation during vascular development [110]. Under normal conditions, the contact of the initial cohort of SMC progenitors with endothelial cells via N-cadherin engages cell surface molecules with signaling activity in both cell types. One such molecule on endothelial cells is the notch ligand jagged-1 [113]. Jagged-1 engages a notch receptor on neural-crest-derived SMC progenitor cells, promoting their differentiation while inducing the expression of jagged-1 in these cells, which then initiates an iterative sequence of notch-receptor-mediated jagged-1 expression in the next layer of SMC progenitors [114]. What stops this lateral induction sequence of notch signaling at the typical number of SMC layers found in different arteries after birth is not yet known but could be directly related to the inhibition of notch signaling in developing artery walls [115,116].

The lineage-mapping studies discussed above [17,52,53,54,55,56] demonstrate that vascular SMCs arising from different embryonic origins do not mix freely in developing artery walls. These non-mixing SMC populations define origin-specific compartments within developing blood vessels into which entry by cells from outside the lineage compartment is prevented. Exactly when and where this encounter takes place is likely to be a function of their relative mobility with the arteries that they invest. For example, the murine left pulmonary artery (PA) at birth consists of an inner layer of endothelial cells, two layers of SMCs, and an outer layer of adventitia [107]. The first layer of PA-SMCs emerges from PDGFRβ-positive mesenchyme surrounding the developing PA wall at around E11.5. As SMC differentiation markers appear in the first layer of SMCs, the expression of PDGFRβ is downregulated in these cells [107]. The second layer of PA-SMCs arises from two sources. The major fraction originates from surrounding PDGFRβ-positive mesenchyme in a sequence much like the first layer. Examination of the nuclear morphology and the orientation of SMC mitotic spindles during PA wall formation showed that during E11.5 to E13.5, the axis of division of SMCs in the first layer was predominantly longitudinal (>75%), thus contributing daughter cells to the growth in length of the PA. At E14.5, however, the axis of division shifts so that the majority of first-layer SMCs divide circumferentially, while cells in the second layer of media and in the adventitia continue to divide in the longitudinal orientation. Therefore, circumferential orientation of the division of first-layer SMCs contributes daughter cells to expand the second layer. Using a single low dose of tamoxifen in *Myh11CreERT2*; *mTmG* mice administered at E11.5 to label individual SMCs, the position of clones originating from inner-layer SMCs could be mapped in the PA wall from E13.5 to E18.5. These experiments showed that individual cells within a clone often dispersed widely from each other, intermixing with unlabeled cells in the same compartment and populating both first-layer and second-layer SMCs. While migrating extensively within the media both longitudinally and circumferentially, labeled SMCs did not enter the adventitia or the intima [107]. These results illustrate two important principles about early vascular development. The first is that cells within nascent blood vessel walls are not stationary but exhibit considerable motility within the developing vessel or primitive vascular network. The second is that barriers, or borders, exist that define compartments into which entry by cells from outside the compartment is prevented. As described in a previous section, compartments and the borders that define them are fundamental to tissue morphogenesis, and it is no surprise to also find them in developing blood vessels.

## 7. The Role of Hemodynamics in PAA Remodeling

Like most developing blood vessels, the morphogenesis of the pharyngeal arch artery complex is highly sensitive to blood flow variations. These blood flow variations can arise from a number of primary causes, including the normal growth of the heart as well as defects originating in the heart, valves, outflow tract, or pharyngeal arch arteries themselves. For example, the *shrunken head* (*shru*) mutation was identified in a forward genetic screen in mice and found to be localized within the *titin* gene [10]. *Shru* produced a hypomorphic allele, and embryos were defective in development of myocardial cells, as revealed by greatly reduced numbers of myofibrils in these cells, reduced cardiac contractile activity, and the pronounced delay in the onset of circulation through the developing vascular system. *Shru* mutants had little or no blood flow from E8.5 to E9.5 and then exhibited a weak initiation of blood flow from E9.5 to embryonic death at E11.5 [10]. A variety of embryonic and extra-embryonic vascular defects were observed in *shru* mutant embryos, including defective endothelial cell–cell junctions, abnormal endothelial cell protrusions, intermittent lack of lumen formation, extensive areas of hypoxia, and increased *VEGFA* gene expression. As titin is expressed in cardiac myocytes but not in endothelial cells, the vascular defects in *shru* embryos are most likely indirect and result from greatly reduced or absent blood flow through the developing vascular system during angiogenesis, plexus reorganization, and PAA remodeling stages. The authors conclude that fluid biomechanical forces produced from the very onset of cardiac contractions are required to orchestrate multiple aspects of endothelial structure and function as well as vascular network formation and remodeling [10]. It will be of interest to learn if the formation or position of SMC-origin-specific borders is disrupted in *shru* embryos.

The outcome of PAA remodeling is also a reflection of the overall control of left–right asymmetry (laterality) during embryonic development [104,117,118]. Control of laterality for internal organs is a function of nodal signaling [117]. Nodal induces the expression of PITX2, a transcription factor that controls left–right asymmetry in cardiac development and aortic arch remodeling [117,118,119]. The asymmetric expression of *Pitx2* is controlled by the asymmetric enhancer element (ASE) in the *Pitx2* locus [117,120]. Mice lacking the ASE develop right isomerism, cardiac developmental defects, and randomized laterality of PAAs [104,120]. While asymmetry of 6th PAA remodeling is randomized in mice lacking the ASE, the 4th PAA was not affected [104]. Laterality of the aortic arch is also randomized in these mice and is dependent upon which side of the paired 6th PAAs undergoes regression. In wild-type embryos, echocardiography studies showed that at E11.5, blood flow through the left dorsal aorta was similar to that in the right dorsal aorta, whereas by E12.0, blood flow was significantly higher in the left compared to the right-side dorsal aorta [121]. Surprisingly, *Pitx2* is not expressed in or near the 6th PAA or the dorsal aorta. Rather, it is strongly expressed in the second heart field, the myocardium, and the left wall of the outflow tract [104,118]. In normal heart development, the formation of the spiral septum of the outflow tract aligns the ventricles with the great arteries, and the rotation of the arterial pole produces changes in the right 6th arch artery structure that reduce blood flow and favor regression of the right side [86]. In ASE-deficient mice, defects in outflow tract morphogenesis result in altered blood flow distribution to the right and left sides of the 6th PAA, leading to randomized laterality of the 6th PAA [104,105,120]. These studies show how closely related the rotational development of the outflow tract is to the normal asymmetric remodeling of the 6th PAA and ultimate formation of the DA. As indicated above for *shru* embryos, it will be interesting to learn if the position of the SMC-origin-dependent border is disrupted if the blood flow distribution through the right versus left 6th PAAs is altered due to defects in cardiac activity, valve function, or outflow tract development.

## 8. Role of Hemodynamics in Vascular Smooth Muscle Cell Investment

As discussed above, PAA remodeling is highly sensitive to changes in blood flow. Evidence from multiple experimental models shows that the investment of nascent endothelial tubes with mural cells (SMCs and pericytes) is also a blood-flow-responsive process [122,123,124]. The release of PDGFB, among other factors, by endothelial cells is stimulated by increased fluid shear stress acting on the endothelial cell surface [125,126]. The abluminal secretion of PDGFB [127] and the activation of nearby PDGFRβ-expressing SMC progenitor cells are key steps in the investment process [108,110,111,128,129]. The effects of PDGFRβ signaling involve the stimulation of SMC progenitor cell chemotaxis [126], migration [125], and localized cell proliferation in vivo [111]. Further analysis suggests two models for the investment process: (a) the de novo formation of SMCs from surrounding undifferentiated mesenchymal progenitor cells, and (b) the migration of SMCs from a pre-existing pool of SMCs usually upstream of the site of investment [111,122,124,128,129,130,131]. One question that is not addressed by these previous studies is how SMC investment proceeds at specific sites in the vascular system where SMCs arising from two different embryonic lineages are involved. What cell dynamics occur when cardiac neural-crest-derived SMCs and paraxial-mesoderm- or second-heart-field-derived SMCs encounter each other during the assembly of the vessel wall? In any case, investment models must account for the apparent lack of mixing at SMC-origin-dependent borders [17,52,132].

## 9. Interactions Between Different Types of Vascular SMC Progenitors

The investment of PAAs with SMCs exhibits the ability to compensate for defects in the number of available CNC-derived SMC progenitor cells. Alexander et al. reported that in mice made Smad4-deficient in the CNC-lineage using Sox10iCre mice, most of the CNC-derived SMC progenitors in the pharyngeal arch mesenchyme were lost due to cell death [133]. In the absence of this dominant source of PAA SMCs, a non-CNC source of locally available SMC progenitors was observed to rescue the investment process so that the 4th and 6th PAAs developed and remodeled normally. Although not tested directly, it is reasonable to assume that this non-CNC source of SMC progenitors was second-heart-field-derived progenitor cells, which are also present in the PA mesenchyme [133]. This finding suggests the possibility that CNC-derived SMC progenitors normally suppress the SMC fate of available non-CNC progenitors, providing a glimpse of the normal cross-talk between SMCs of different embryonic origins at compartment borders. If investment itself is a blood-flow-dependent process, then the communication between SMC progenitors as well as the final structure or position of SMC-origin-dependent borders may also be influenced by blood flow dynamics.

## 10. Smooth Muscle Compartment Boundary Model for CoA

**Figure 4:**
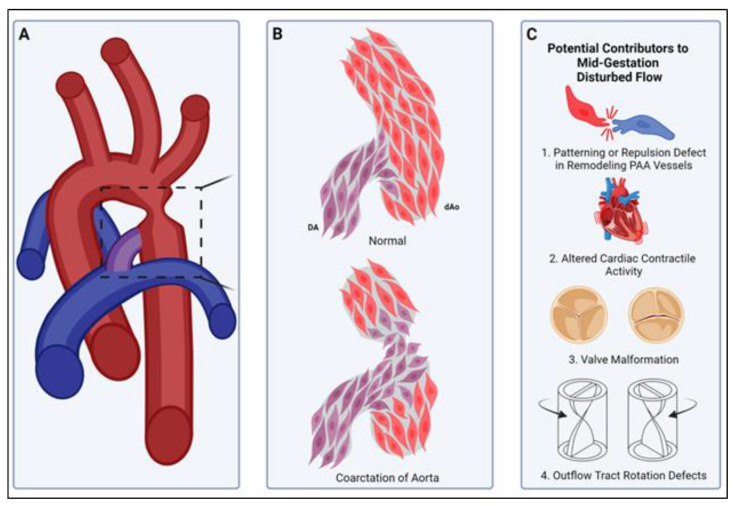
A smooth muscle compartment boundary model of CoA. (**A**) Illustration of a common site of aortic coarctation where the ductus arteriosus (purple) joins the descending thoracic aorta (red). Boxed area is illustrated in more detail in panel (**B**). (**B**, **top**) Normally, cardiac neural-crest-derived DA-SMCs (DA, purple) and paraxial-mesoderm-derived descending Ao-SMCs (dAo, red) fail to mix, and a compartment boundary is formed at their interface (see insertion junction, Figure 3). (**B**, **bottom**) In coarctation of the aorta, loss of the compartment boundary results in DA-SMCs (DA, purple) invading the aortic media (dAo, red), producing an aortic constriction upon birth of the neonate. (**C**) Potential mechanisms for the pathogenesis of CoA. The normal DA-SMC interface with Ao-SMCs is established during SMC investment of pharyngeal arch artery (PAA) walls in mid-gestation (see, Figure 2). The remodeling of the paired PAAs is highly sensitive to changes in blood flow rate and flow distribution through the right and left sides of the PAAs [10,15,104,105]. Either disruptions in SMC cell–cell interactions that normally prevent mixing across SMC-origin-dependent compartment boundaries (C1) or defects in cardiac contractile activity (C2), valve morphogenesis (C3), or outflow tract formation/rotation (C4) can alter the hemodynamic shear stress distribution during PAA remodeling. We propose that the formation of the normal DA-Ao SMC interface (**B**) is also a flow-sensitive process during PAA remodeling, and disturbed patterns of SMC investment during PAA remodeling can produce CoA observed in neonates.

We propose a vascular SMC compartment boundary model for the pathogenesis of CoA. This model suggests that CoA is the consequence of a failure to form a SMC-origin-specific compartment border in its normal position at the ductal ostia (Figure 3 and Figure 4). We propose that disturbed blood flow during remodeling of the 6th PAA, either due to direct changes (somatic genetic or epigenetic) in the 6th PAA cells themselves or indirect hemodynamic effects due to altered cardiac contractile activity, cardiac valve structure or function or outflow tract defects, could lead to an altered position or absence of the DA-SMC junction with AO-SMCs at the ductal ostia, thus allowing DA-SMCs to invade the aortic wall (Figure 4B,C). This would explain the localization of a majority of CoA lesions to the ductal–aorta junction and the clinical observation that CoA can be detected by fetal echo before birth [11,12]. It would also explain the frequent co-occurrence of CoA with bicuspid aortic valve and other cardiac congenital defects that would disturb blood flow during PAA remodeling [1,10,100,134,135]. This model for the origin for CoA raises important new questions: (1) if the DA-AO compartment border is the consequence of an interface of SMCs with different identities, similar to other developmental borders, then what molecules and mechanisms are responsible for the specification of the distinct identities of DA-SMCs vs. AO-SMCs during vascular development? (2) what cell surface molecules and signaling pathways normally act to prevent the mixing of DA-SMCs and AO-SMCs at the ductal–aorta interface? and (3) what mechanisms disrupt the expression, function, or localization of these border-forming molecules during the pathogenesis of CoA? Although we have focused on a role for the loss of origin-specific SMC compartment boundaries in our model for the pathogenesis of CoA, we emphasize that additional factors including genetic, environmental, and metabolic regulation act together with hemodynamic forces in a complex pattern of interactions to produce CoA observed at birth. Given that vascular SMC origins are far more diverse than just CNC and SHF, the answers to the questions raised above and the molecular insights gained may provide a better understanding of vascular pathologies at other sites within the mosaic vascular system as a whole.

## Data Availability

No new data were created or analyzed in this study.

## References

[1] Hoffman J.I., Kaplan S. (2002). The incidence of congenital heart disease. J. Am. Coll. Cardiol..

[2] Mai C.T., Isenburg J.L., Canfield M.A., Meyer R.E., Correa A., Alverson C.J., Lupo P.J., Riehle-Colarusso T., Cho S.J., Aggarwal D. (2019). National population-based estimates for major birth defects, 2010–2014. Birth Defects Res..

[3] Tagariello A., Breuer C., Birkner Y., Schmidt S., Koch A.M., Cesnjevar R., Ruffer A., Dittrich S., Schneider H., Winterpacht A. (2012). Functional null mutations in the gonosomal homologue gene TBL1Y are associated with non-syndromic coarctation of the aorta. Curr. Mol. Med..

[4] Freylikhman O., Tatarinova T., Smolina N., Zhuk S., Klyushina A., Kiselev A., Moiseeva O., Sjoberg G., Malashicheva A., Kostareva A. (2014). Variants in the NOTCH1 gene in patients with aortic coarctation. Congenit. Heart Dis..

[5] Moosmann J., Uebe S., Dittrich S., Rüffer A., Ekici A., Toka O. (2015). Novel loci for non-syndromic coarctation of the aorta in sporadic and familial cases. PLoS ONE.

[6] Sanchez-Castro M., Pichon O., Briand A., Poulain D., Gournay V., David A., Le Caignec C. (2015). Disruption of the SEMA3D gene in a patient with congenital heart defects. Hum. Mutat..

[7] Bjornsson T., Thorolfsdottir R.B., Sveinbjornsson G., Sulem P., Norddahl G.L., Helgadottir A., Gretarsdottir S., Magnusdottir A., Danielsen R., Sigurdsson E.L. (2018). A rare missense mutation in MYH6 associates with non-syndromic coarctation of the aorta. Eur. Heart J..

[8] Donadille B., Christin-Maitre S. (2021). Heart and Turner syndrome. Ann. Endocrinol..

[9] Hiruma T., Nakajima Y., Nakamura H. (2002). Development of pharyngeal arch arteries in early mouse embryo. J. Anat..

[10] May S.R., Stewart N.J., Chang W., Peterson A.S. (2004). A *Titin* mutation defines roles for circulation in endothelial morphogenesis. Dev. Biol..

[11] Franklin O., Burch M., Manning N., Sleeman K., Gould S., Archer N. (2002). Prenatal diagnosis of coarctation of the aorta improves survival and reduces morbidity. Heart.

[12] Houshmandi M.M., Eckersley L., Fruitman D., Mills L., Power A., Hornberger L.K. (2021). Fetal diagnosis is associated with improved perioperative condition of neonates requiring surgical intervention for coarctation. Pediatr. Cardiol..

[13] Waldo K.L., Kirby M.L. (1993). Cardiac neural crest contribution to the pulmonary artery and sixth aortic arch artery complex in chick embryos aged 6 to 18 days. Anat. Rec..

[14] Bökenkamp R., DeRuiter M.C., van Munsteren C., Gittenberger-de Groot A.C. (2010). Insights into the pathogenesis and genetic background of patency of the ductus arteriosus. Neonatology.

[15] Gittenberger-de Groot A.C., Peterson J.C., Wisse L.J., Roest A.A.W., Poelmann R.E., Bökenkamp R., Elzenga N.J., Kaxekamp M., Bartelings M.M., Jongbloed M.R.M. (2020). Pulmonary ductal coarctation and left pulmonary artery interruption; pathology and role of neural crest and second heart field during development. PLoS ONE.

[16] Wang X., Chen D., Chen K., Jubran A., Ramirez A., Astrof S. (2017). Endothelium in the pharyngeal arches 3,4 and 6 is derived from the second heart field. Dev. Biol..

[17] Jiang X., Rowitch D.H., Soriano P., McMahon A.P., Sucov H.M. (2000). Fate of the mammalian cardiac neural crest. Development.

[18] Wasteson P., Johansson B.R., Jukkola T., Breuer S., Akyürek L.M., Partanen J., Lindahl P. (2008). Developmental origin of smooth muscle cells in the descending aorta in mice. Development.

[19] Elzenga N.J., Gittenberger-de Groot A.C. (1983). Localised coarctation of the aorta. An age dependent spectrum. Br. Heart J..

[20] Rabinovitch M. (1996). Cell-extracellular matrix interactions in the ductus arteriosus and perinatal pulmonary circulation. Semin. Perinatol..

[21] Bentley R.E.T., Hindmarch C.C.T., Dunham-Snary K.J., Snetsinger B., Mewburn J.D., Thébaud A., Lima P.D., Thébaud B., Archer S.L. (2021). The molecular mechanisms of oxygen-sensing in human ductus arteriosus smooth muscle cells: A comprehensive transcriptome profile reveals a central role for mitochondria. Genomics.

[22] Dahmann C., Basler K. (1999). Compartment boundaries: At the edge of development. Trends Genet..

[23] Brand-Saberi B., Christ B. (2000). Evolution and development of distinct cell lineages derived from somites. Curr. Top. Dev. Biol..

[24] Groves A.K., LaBonne C. (2014). Setting appropriate boundaries: Fate, patterning and competence at the neural plate border. Dev. Biol..

[25] Fagotto F. (2014). The cellular basis of tissue separation. Development.

[26] Wang J., Dahmann C. (2020). Establishing compartment boundaries in Drosophila wing imaginal discs: An interplay between selector genes, signaling pathways and cell mechanics. Semin. Cell Dev. Biol..

[27] Fagotto F. (2020). Cell sorting at embryonic boundaries. Semin. Cell Dev. Biol..

[28] Maves L., Jackman W., Kimmel C.B. (2002). FGF3 and FGF8 mediate a rhombomere 4 signaling activity in the zebrafish hindbrain. Development.

[29] Cooke J.E., Kemp H.A., Moens C.B. (2005). EphA4 is required for cell adhesion and rhombomere-boundary formation in the zebrafish. Curr. Biol..

[30] Kiecker C., Lumsden A. (2005). Compartments and their boundaries in vertebrate brain development. Nat. Rev. Neurosci..

[31] Tannahill D., Cook G.M., Keynes R.J. (1997). Axon guidance and somites. Cell Tissue Res..

[32] Takahashi Y., Koizumi K., Takagi A., Kitajima S., Inoue T., Koseki H., Saga Y. (2000). Mesp2 initiates somite segmentation through the Notch signalling pathway. Nat. Genet..

[33] Watanabe T., Sato Y., Saito D., Tadakoro R., Takahashi Y. (2009). EphrinB2 coordinates the formation of a morphological boundary and cell epithelialization during somite segmentation. Proc. Natl. Acad. Sci. USA.

[34] Franco D., Meilhac S.M., Christoffels V.M., Kispert A., Buckingham M., Kelley R.G. (2006). Left and right ventricular contributions to the formation of the interventricular septum in the mouse heart. Dev. Biol..

[35] Kathiriya I.S., Dominguez M.H., Ro K.S., Muncie-Vasic J.M., Devine W.P., Hu K.M., Hota S.K., Garay B.I., Quintero D., Goyal P. (2024). A disrupted compartment boundary underlies abnormal cardiac patterning and congenital heart defects. bioRxiv.

[36] Kimmel R.A., Turnbull D.H., Blanquet V., Wurst W., Loomis C.A., Joyner A.L. (2000). Two lineage boundaries coordinate vertebrate apical ectodermal ridge formation. Genes Dev..

[37] Qiu Q., Chen H., Johnson R.L. (2009). Lmx1b-expressing cells in the mouse limb bud define a dorsal mesenchymal lineage compartment. Genesis.

[38] Morata G., Herrera S.C. (2016). Cell reprogramming during regeneration in Drosophila: Transgression of compartment boundaries. Curr. Opin. Genet. Dev..

[39] Sharrock T.E., Sanson B. (2020). Cell sorting and morphogenesis in early *Drosophila* embryos. Semin. Cell Dev. Biol..

[40] Lawrence P.A. (1992). The Making of a Fly: The Genetics of Animal Design.

[41] Worley M.I., Setiawan L., Hariharan I.K. (2012). Regeneration and transdetermination in *Drosophila* imaginal discs. Annu. Rev. Genet..

[42] Townes P.L., Holtfreter J. (1955). Directed movements and selective adhesion of embryonic amphibian cells. J. Exp. Zool..

[43] Nose A., Nagafuchi A., Takeichi M. (1988). Expressed recombinant cadherins mediate cell sorting in model systems. Cell.

[44] Steinberg M.S. (2007). Differential adhesion in morphogenesis: A modern view. Curr. Opin. Genet. Dev..

[45] Brodland G.W. (2002). The differential interfacial tension hypothesis (DITH): A comprehensive theory for the self-rearrangement of embryonic cells and tissues. J. Biomech. Eng..

[46] Wang H.U., Xhen Z.F., Anderson D.J. (1998). Molecular distinction and angiogenic interaction between embryonic arteries and veins revealed by ephrin-B2 and its receptor Eph-B4. Cell.

[47] Gale N.W., Baluk P., Pan L., Kwan M., Holash J., DeChiara T.M., McDonald D.M., Yancopoulos G.D. (2001). Ephrin-B2 selectively marks arterial vessels and neovascularization sites in the adult, with expression in both endothelial and smooth muscle cells. Dev. Biol..

[48] Adams R.H., Eichmann A. (2010). Axon guidance molecules in vascular patterning. Cold Spring Harb. Perspect. Biol..

[49] Shin D., Garcia-Cardena G., Hayashi S., Gerety S., Asahara T., Stavrakis G., Isner J., Folkman J., Gimbrone M.A., Anderson D.J. (2001). Expression of ephrinB2 identifies a stable genetic difference between arterial and venous vascular smooth muscle as well as endothelial cells, and marks subsets of microvessels at sites of adult neovascularization. Dev. Biol..

[50] Finney A.C., Orr A.W. (2018). Guidance molecules in vascular smooth muscle. Front. Physiol..

[51] Tian X., Hu T., Je L., Zhang H., Huang X., Poelmann R.E., Liu W., Yang Z., Yan Y., Pu W.T. (2013). Peritruncal coronary endothelial cells contribute to proximal coronary artery stems and their aortic orifices in the mouse heart. PLoS ONE.

[52] Majesky M.W. (2007). Developmental basis of vascular smooth muscle diversity. Arterioscler. Thromb. Vasc. Biol..

[53] Passman J.N., Dong X.R., Wu S.P., Maguire C.T., Hogan K.A., Bautch V.L., Majesky M.W. (2008). A sonic hedgehog signaling domain in the arterial adventitia supports resident Sca1+ smooth muscle progenitor cells. Proc. Natl. Acad. Sci. USA.

[54] Sawada H., Rateri D.L., Moorleghen J.J., Majesky M.W., Daugherty A. (2017). Smooth muscle cells derived from the second heart field and cardiac neural crest reside in spatially distinct domains in the media of the ascending aorta—Brief report. Arterioscler. Thromb. Vasc. Biol..

[55] Lin C.J., Hunkins B., Roth R., Lin C.Y., Wagenseil J.E., Mecham R.P. (2021). Vascular smooth muscle cell subpopulations and neointimal formation in mouse models of elastin insufficiency. Arterioscler. Thromb. Vasc. Biol..

[56] Sawada H., Katsumata y Higashi H., Zhang C., Li Y., Morgan S., Lee L.H., Singh S.A., Chen J.Z., Franklin M.K., Moorleghen J.J. (2022). Second heart field-derived cells contribute to angiotensin II-mediated ascending aortopathies. Circulation.

[57] Pedroza A.J., Dalal A.R., Shad RYokoyama N., Nakamura K., Cheng P., Wirka R.C., Mitchel O., Baiocchi M., Hiesinger W., Quertermous T. (2022). Embryologic origin influences smooth muscle cell phenotypic modulation signatures in murine Marfan syndrome aortic aneurysm. Arterioscler. Thromb. Vasc. Biol..

[58] Schüle K.M., Probst S. (2025). Epigenetic control of cell identities from epiblast to gastrulation. FEBS J..

[59] John R.M., Rougeulle C. (2018). Developmental epigenetics: Phenotype and the flexible epigenome. Front. Cell Dev. Biol..

[60] Nakamura K., Dalal A.R., Yokoyama N., Pedroza A.J., Kusadokoro S., Mitchel O., Gilles C., Masoudian B., Leipzig M., Casey K.M. (2023). Lineage-specific induced pluripotent stem cell-derived smooth muscle cell modeling predicts integrin alpha-v antagonism reduces aortic root aneurysm formation in Marfan syndrome mice. Arterioscler. Thromb. Vasc. Biol..

[61] Basler K., Struhl G. (1994). Compartment boundaries and the control of Drosophila limb pattern by hedgehog protein. Nature.

[62] Lawrence P.A., Struhl G. (1996). Morphogens, compartments, and pattern: Lessons from Drosophila?. Cell.

[63] Tessier-Lavigne M., Goodman C.S. (1996). The molecular biology of axon guidance. Science.

[64] Zuhdi N., Ortega B., Giovannone D., Ra H., Reyes M., Asención V., McNicoll I., Ma L., de Bellard M.E. (2015). Slit molecules prevent entrance of trunk neural crest cells in developing gut. Int. J. Dev. Neurosci..

[65] Lepore J.J., Mericko P.A., Cheng L., Lu M.M., Morrisey E.E., Parmacek M.S. (2006). GATA-6 regulates semaphorin 3C and is required in cardiac neural crest for cardiovascular morphogenesis. J. Clin. Investig..

[66] High F., Epstein J.A. (2007). Signaling pathways regulating cardiac neural crest migration and differentiation. Novartis Found. Symp..

[67] Toyofuku T., Yoshida J., Sugimoto T., Yamamoto M., Makino N., Takamatsu H., Takegahara N., Suto D., Hori M., Fujisawa H. (2008). Repulsive and attractive semaphorins cooperate to direct the navigation of cardiac neural crest cells. Dev. Biol..

[68] Scholl A.M., Kirby M.L. (2009). Signals controlling neural crest contributions to the heart. Wiley Interdiscip. Rev..

[69] Schulz Y., Wehner P., Optiz L., Salinas-Riester G., Bongers E.M.H.F., van Ravenswaaij-Arts C.M.A., Wincent J., Schoumans J., Kohlhase J., Borchers A. (2014). CHD7, the gene mutated in CHARGE syndrome, regulates genes involved in neural crest cell guidance. Hum. Genet..

[70] Kodo K., Shibata S., Miyagawa-Tomita S., Ong S.G., Takahashi H., Kume T., Okano H., Matsuoka R., Yamagishi H. (2017). Regulation of Sema3c and the interaction between cardiac neural crest and second heart field during outflow tract development. Sci. Rep..

[71] Schussler O., Gharibeh L., Mootoosamy P., Murith N., Tien V., Rougemont A.L., Sologashvili T., Suuronen E., Lecarpentier Y., Ruel M. (2021). Cardiac neural crest cells: Their rhombomeric specification, migration, and association with heart and great vessel anomalies. Cell. Mol. Neurobiol..

[72] Becker S.F.S., Mayor R., Kashef J. (2013). Cadherin-11 mediates contact inhibition of locomotion during Xenopus neural crest cell migration. PLoS ONE.

[73] Carmona-Fontaine C., Matthews H.K., Kuriyama S., Moreno M., Dunn G.A., Parsons M., Stern C.D., Mayor R. (2008). Contact inhibition of locomotion in vivo controls neural crest directional migration. Nature.

[74] Mayor R., Carmona-Fontaine C. (2010). Keeping in touch with contact inhibition of locomotion. Trends Cell Biol..

[75] Theveneau E., Marchant L., Kuriyama S., Gull M., Moepps B., Parsons M., Mayor R. (2010). Collective chemotaxis requires contact-dependent cell polarity. Dev. Cell.

[76] Willecke M., Hamaratoglu F., Sansores-Garcia L., Tal C., Halder G. (2008). Boundaries of Dachsous Cadherin activity modulate the Hippo signaling pathway to induce cell proliferation. Proc. Natl. Acad. Sci. USA.

[77] Huang J., Cheng L., Li J., Chen M., Zhou D., Lu M.M., Proweller A., Epstein J.A., Parmacek M.S. (2008). Myocardin regulates expression of contractile genes in smooth muscle cells and is required for closure of the ductus arteriosus in mice. J. Clin. Investig..

[78] Yancopoulos G.D., Klagsbrun M., Folkman J. (1998). Vasculogenesis, angiogenesis, and growth factors: Ephrins enter the fray at the border. Cell.

[79] Wu M.F., Liao C.Y., Wang L.Y., Chang J.T. (2017). The role of Slit-Robo signaling in the regulation of tissue barriers. Tissue Barriers.

[80] Waldo K., Hutson M., Ward C., Zdanowicz M., Stadt H., Kumiski D., Abu-Issa R., Kirby M. (2005). Secondary heart field contributes myocardium and smooth muscle to the arterial pole of the developing heart. Dev. Biol..

[81] Topouzis S., Majesky M.W. (1996). Smooth muscle lineage diversity in the chick embryo. Two types of aortic smooth muscle cell differ in growth and receptor-mediated transcriptional responses to transforming growth factor-beta. Dev. Biol..

[82] MacFarlane E.G., Parker S.J., Shin J.Y., Kang B.E., Ziegler S.G., Creamer T.J., Bagirzadeh R., Bedja D., Chen Y., Calderon J.F. (2019). Lineage-specific events underlie aortic root aneurysm pathogenesis in Loeys-Dietz syndrome. J. Clin. Investig..

[83] Shukla S., Jana S., Sanford N., Lee C.Y., Liu L., Cheng P., Quertermous T., Dichek D.A. (2025). Single-cell transcriptomics identifies selective lineage-specific regulation of genes in aortic smooth muscle cells in mice. Arterioscler. Thromb. Vasc. Biol..

[84] Weldy C.S., Cheng P.P., Guo W., Pedroza A.J., Dalal A.R., Worssam M.D., Sharma D., Nguyen T., Kundu R., Fischbein M.P. (2022). The epigenomic landscape of single vascular cells reflects developmental origin and identifies disease risk loci. bioRxiv.

[85] Jaffe M., Sesti C., Washington I.M., Du L., Dronadula N., Chin M.T., Stolz D.B., Davis A.C., Dichek D.A. (2012). Transforming growth factor-β signaling in myogenic cells regulates vascular morphogenesis, differentiation, and matrix synthesis. Arterioscler. Thromb. Vasc. Biol..

[86] Kirby M.L. (2007). Cardiac Development.

[87] Le Noble F., Moyon D., Pardanaud L., Yuan L., Djonov V., Matthijsen R., Bréant C., Fluery V., Eichmann A. (2004). Flow regulates arterial-venous differentiation in the chick embryo yolk sac. Development.

[88] Lucitti J.L., Jones E.A.V., Huang C., Chen J., Fraser S.E., Dickinson M.E. (2007). Vascular remodeling of the mouse yolk sac requires hemodynamic force. Development.

[89] Kowalski W.J., Dur O., Wang Y., Patrick M.J., Tinney J.P., Keller B.B., Pekkan K. (2013). Critical transitions in early embryonic aortic arch patterning and hemodynamics. PLoS ONE.

[90] Langille B.L. (1996). Arterial remodeling: Relation to hemodynamics. Can. J. Physiol. Pharmacol..

[91] Baeyens N., Bandopadhyay C., Coon B.G., Yun S., Schwartz M.A. (2016). Endothelial fluid shear stress sensing in vascular health and disease. J. Clin. Investig..

[92] Langille B.L. (2001). Morphologic responses of endothelium to shear stress: Reorganization of the adherens junction. Microcirculation.

[93] Bi W., Drake C.J., Schwarz J.J. (1999). The transcription factor *Mef2c-*null mouse exhibits complex vascular malformations and reduced cardiac expression of angiopoietin-1 and VEGF. Dev. Biol..

[94] Isogai S., Lawson N.D., Torrealday S., Horiguchi M., Weinstein B.M. (2009). Angiogenic network formation in the developing vertebrate trunk. Development.

[95] Udan R.S., Vadakkan T.J., Dickinson M.E. (2013). Dynamic responses of endothelial cells to changes in blood flow during vascular remodeling of the mouse yolk sac. Development.

[96] Tzima E., Irani-Tehrani M., Klosses W.B., Dejana E., Schultz D.A., Engelhardt B., Cao G., DeLisser H., Schwartz M.A. (2005). A mechanosensory complex that mediates the endothelial cell response to fluid shear stress. Nature.

[97] Jones E.A.V., le Noble F., Eichmann A. (2006). What determines blood vessel structure? Genetic prespecification vs hemodynamics. Physiology.

[98] Mehta V., Pang K.L., Rozbesky D., Nather K., Keen A., Lachowski D., Kong Y., Karia D., Ameismeier M., Huang J. (2020). The guidance receptor plexin D1 is a mechanosensor in endothelial cells. Nature.

[99] Jung B., Obinata H., Galvani S., Mendelson K., Ding B., Skoura A., Kinzel B., Brinkmann V., Rafii S., Evans T. (2012). Flow-regulated endothelial S1P receptor-1 signaling sustains vascular development. Dev. Cell.

[100] Li J., Hou B., Tumova S., Muraki K., Bruns A., Ludlow M.J., Sedo A., Hyman A.J., McKeown L., Young R.S. (2014). Piezo1 integration of vascular architecture with physiological force. Nature.

[101] Nauli S.M., Kawanabe Y., Kaminski J.J., Pearce W.J., Ingber D.E., Zhou J. (2008). Endothelial cilia are fluid shear sensors that regulate calcium signaling and nitric oxide production through polycystin-1. Circulation.

[102] Wang Y., Dur O., Patrick M.J., Tinney J.P., Tobita K., Keller B.B., Pekkan K. (2009). Aortic arch morphogenesis and flow modeling in the chick embryo. Ann. Biomed. Eng..

[103] Kowalski W.J., Pekkan K., Tinney J.P., Keller B.K. (2014). Investigating developmental cardiovascular biomechanics and the origins of congenital heart disease. Front. Physiol..

[104] Yashiro K., Shiratori H., Hamada H. (2007). Haemodynamics determined by a genetic programme govern asymmetric development of the aortic arch. Nature.

[105] Snider P., Conway S.J. (2007). Developmental biology: The power of blood. Nature.

[106] Karakaya C., Goktas S., Celik M., Kowalski W.J., Keller B.B., Pekkan K. (2018). Asymmetry in mechanosensitive gene expression during aortic arch morphogenesis. Sci. Rep..

[107] Greif D.M., Kumar M., Lighthouse J.K., Hum J., An A., Ding L., Red-Horse K., Espinoza F.H., Olson L., Offermanns S. (2012). Radial construction of an arterial wall. Dev. Cell.

[108] Siekmann A.F. (2023). Biology of vascular mural cells. Development.

[109] Stratman A.M., Burns M.C., Farrelly O.M., Davis A.E., Li W., Pham V.N., Castranova D., Yano J.J., Goddard L.M., Nguyen O. (2020). Chemokine mediated signaling within arteries promotes vascular smooth muscle cell recruitment. Commun. Biol..

[110] Lee J., Goeckel M.E., Levitas A., Colijn S., Shin J., Hindes A., Mun G., Burton Z., Chintalapati B., Yin Y. (2024). CXCR3-CXCL11 signaling restricts angiogenesis and promotes pericyte recruitment. Arterioscler. Thromb. Vasc. Biol..

[111] Hellström M., Kalén M., Lindahl P., Abramsson A., Betsholtz C. (1999). Role of PDGF-B and PDGFR-beta in recruitment of vascular smooth muscle cells and pericytes during embryonic blood vessel formation in the mouse. Development.

[112] Ando K., Shih Y.H., Ebarasi L., Grosse A., Portman D., Chiba A., Mattonet K., Gerri C., Stainier D.Y.R., Mochizuki N. (2021). Conserved and context-dependent roles for pdgfrb signaling in zebrafish mural cell development. Dev. Biol..

[113] High F.A., Lu M.M., Pear W.S., Loomes K.M., Kaestner K.H., Epstein J.A. (2008). Endothelial expression of the Notch ligand Jagged1 is required for vascular smooth muscle development. Proc. Natl. Acad. Sci. USA.

[114] Manderfield L.J., High F.A., Engleka K.A., Liu F., Li L., Rentschler S., Epstein J.A. (2012). Notch activation of Jagged1 contributes to the assembly of the arterial wall. Circulation.

[115] Kangsamaksin T., Tattersall I.W., Kitajewski J. (2014). Notch functions in development and tumor angiogenesis by diverse mechanisms. Biochem. Soc. Trans..

[116] Sen P., Ghosh S.S. (2023). Intricate Notch signaling dynamics in therapeutic realms of cancer. ACS Pharmacol. Transl. Sci..

[117] Hamada H., Meno C., Watanabe D., Saijoh Y. (2002). Establishment of vertebrate left-right asymmetry. Nat. Rev. Genet..

[118] Liu C., Liu W., Palie J., Lu M.F., Brown N.A., Martin J.F. (2002). Pitx2c patterns anterior myocardium and aortic arch vessels and is required for local cell movement into atrioventricular cushions. Development.

[119] Hill M.C., Kadow Z.A., Li L., Tran T.T., Wythe J.D., Martin J.F. (2019). A cellular atlas of *Pitx2*-dependent cardiac development. Development.

[120] Shiratori H., Sakuma R., Watanabe M., Hashiguchi H., Michida K., Sakai Y., Nishino J., Saijoh Y., Whitman M., Hamada H. (2001). Two-step regulation of left-right asymmetric expression of Pitx2: Initiation by nodal signaling and maintenance by Nkx2. Mol. Cell.

[121] Phoon C.K., Aristizabal O., Turnbull D.H. (2000). 40 MHz doppler characterization of umbilical and dorsal aortic blood flow in the early mouse embryo. Ultrasound Med. Biol..

[122] Chen X., Gays D., Milia C., Santoro M.M. (2017). Cilia control vascular mural cell recruitment in vertebrates. Cell Rep..

[123] Padget R.L., Mohite S.S., Hoog T.G., Justis B.S., Green B.E., Udan R.S. (2019). Hemodynamic force is required for vascular smooth muscle cell recruitment to blood vessels during mouse embryonic development. Mech. Dev..

[124] Cheng S., Xia I.F., Wanner R., Abello J., Stratment A.N., Nicoli S. (2024). Hemodynamics regulate spatiotemporal artery muscularization in the developing circle of Willis. eLife.

[125] Palumbo R., Gaetano C., Antonini A., Pompilio G., Bracco E., Rönnstrand L., Heldin C.H., Capogrossi M.C. (2002). Different effects of high and low shear stress on platelet-derived growth factor isoform release by endothelial cells: Consequences for smooth muscle cell migration. Arterioscler. Thromb. Vasc. Biol..

[126] Dardik A., Yamashita A., Aziz F., Asada H., Sumpio B.E. (2005). Shear stress-stimulated endothelial cells induce smooth muscle cell chemotaxis via platelet-derived growth factor-BB and interleukin-1alpha. J. Vasc. Surg..

[127] Zerwes H.G., Risau W. (1987). Polarized secretion of a platelet-derived growth factor-like chemotactic factor by endothelial cells in vitro. J. Cell Biol..

[128] Stratman A.N., Pezoa S.A., Farrelly O.M., Castranova D., Dye L.E., Butler M.G., Sidik H., Talbot W.S., Weinstein B.M. (2017). Interactions between mural cells and endothelial cells stabilize the developing zebrafish dorsal aorta. Development.

[129] Leonard E.V., Figueroa R.J., Bussmann J., Lawson N.D., Amigo J.D., Siekmann A.F. (2022). Regenerating vascular mural cells in zebrafish fin blood vessels are not derived from pre-existing mural cells and differentially require Pdgfrb signaling for their development. Development.

[130] Grazioli A., Alves C.S., Konstantopoilos K., Yang J.T. (2006). Defective blood vessel development and pericyte/pvSMC distribution in alpha 4 integrin-deficient mouse embryos. Dev. Biol..

[131] Ando K., Fukuhara S., Izumi N., Nakajima H., Fukui H., Kelsh R.N., Mochizuki N. (2016). Clarification of mural cell coverage of vascular endothelial cells by live imaging of zebrafish. Development.

[132] Sinha S., Iyer S., Granata A. (2014). Embryonic origins of human vascular smooth muscle cells: Implications for in vitro modeling and clinical application. Cell. Mol. Life Sci..

[133] Alexander B.E., Zhao H., Astrof S. (2024). SMAD4: A critical regulator of cardiac neural crest fate and vascular smooth muscle development. Dev. Dyn..

[134] Yokoyama U., Ichikawa Y., Minamisawa S., Ishikawa Y. (2017). Pathology and molecular mechanisms of coarctation of the aorta and its association with the ductus arteriosus. J. Physiol. Sci..

[135] Sinning C., Zengin E., Kozlik-Feldman R., Blakenberg S., Rickers C., von Kodolitsch Y., Girdauskas E. (2018). Bicuspid aortic valve and aortic coarctation in congenital heart disease. Cardiovasc. Diagn. Ther..

